# Impacts of Vinyl Group on the Diffusion and Thermal Properties of Branched Silicon-Containing Arylacetylene Resins by Molecular Dynamics Simulations

**DOI:** 10.3390/molecules29235737

**Published:** 2024-12-05

**Authors:** Hui Li, Zijian Sun, Lei Yang, Weihua Zhu

**Affiliations:** Institute for Computation in Molecular and Materials Science, School of Chemistry and Chemical Engineering, Nanjing University of Science and Technology, Nanjing 210094, China; lihui.sun@foxmail.com (H.L.); zijian.sun@njust.edu.cn (Z.S.); yanglei21@njust.edu.cn (L.Y.)

**Keywords:** silicon-containing arylacetylene resins, molecular dynamic simulation, vinyl group, thermal property

## Abstract

Branched-structure polyarylacetylene resins with low curing temperatures and excellent thermal stability are promising candidates for high-performance resin-matrix composites. In this work, the effects of different vinyl/acetylene group ratios on the thermal properties of star-shaped silicon-containing arylacetylene (SSA) resins and branched silicon-containing arylacetylene (BSA) resins were investigated by molecular dynamics simulations. The calculated interaction energies, diffusion behaviors, and glass transition temperatures (*T*_g_) indicated that an increase in the vinyl/acetylene group ratio positively affected the rheological and thermal properties of the branched resins. After the introduction of the vinyl groups, the *T*_g_ value of SSA2 was greater than that of SSA0. An energy decomposition approach (EDA) analysis further confirmed that the involvement of the vinyl groups in the BSA and SSA resins provided larger interaction energies dominated by dispersion effects, leading to better thermal performance. This work may provide us with a deep understanding of the incorporation of vinyl groups in silicon-containing arylacetylene resins.

## 1. Introduction

High-performance resin-matrix composites have a wide range of practical applications in aerospace, electronics, adhesives, and the automotive industry due to their excellent processability, mechanical properties, and thermo-oxidative stability [1,2,3]. In recent years, silicon-containing polyarylacetylene (PSA), as a thermosetting resin, has become an available candidate for high-performance resin-matrix composites [4,5,6]. During the curing process, PSA monomers form a three-dimensional network structure through the alkyne reaction, which exhibits low water absorption, excellent thermal stability, and dielectric properties. Due to the high molecular weight and poor solubility of PSA resins, their development has been hindered by the contradiction between thermal stability and processing performance. When the glass transition temperature of PSA resins with conjugated aromatic structure increases, their processing performance decreases. For example, Huang’s group reported a series of silicon-containing arylacetylene resins with a rigid-rod 2,5-diphenyl-1,3,4-oxadiazole structure [7]. It was found that these resins with low flowability had excellent mechanical and thermal properties, with a 5% decomposition temperature (*T*_d5_) above 450 °C, but were not suitable for resin transfer molding (RTM) processing.

To solve this contradiction, several feasible strategies have been adopted to enhance the rheology of resins: the introduction of various structures into the main chain of the resin (e.g., 2,6-diphenoxypyridine unit [8], side alkoxide [9]) or by incorporating the least polar group (e.g., −S− [10], −CH_3_ [11], −CF_3_ [12]). Li et al. [13] explored the structure–property relationships of PSEF resins by changing the side groups (−H, −CH_3_, −C_6_H_5_, or −CH=CH_2_). It was found that PSEF resin with phenyl substituent exerted a favorable effect on the processing performance, while the PSEF resin with vinyl substituent had better thermal properties. Ma et al. [14] synthesized a series of PSA resins with side aromatic phenyl and phenylacetylene groups (PODSA-2P-MM and PODSA-2E-MM), which exhibited higher heat resistance and lower melting temperature compared with PODSA-MM resin without side groups. This suggests that the side groups play an important role in determining the properties of the resin. On the other hand, the topology of the resin, like branched and hyperbranched structures, is conducive to reducing chain entanglement, thereby resulting in excellent processability and mechanical [15,16]. For example, star-shaped silicon-containing arylacetylene (SSA) resin has a thermal curing temperature of 203 °C, which is much lower than that of linear PSA resin (245 °C) [15]. As expected, a dendritic poly (methylsilane arylacetylene) (DPSA-H) resin with long arms has a tighter stacking network due to its dendritic structure. Cured DPSA-H resins show good heat-resistance in nitrogen with *T*_d5_ of 750 °C [16].

The use of molecular dynamics (MD) simulations is increasing in research on resins and their composites. This is mainly due to the cost advantage and convenience of MD simulations compared with experiments, as well as their ability to effectively reveal the microscopic aspects of resin materials, such as the formation of cross-linked structures at the atomic/molecular scale, and the link between the microstructure and the resin’s properties [17,18,19]. MD simulations are widely used to study the cured structure and predict the properties of high-performance resins, such as pyrolysis behavior, oxidative aging, and rheological and mechanical properties [20,21,22,23]. The calculation and prediction of the properties of epoxy and other thermosetting resins have been experimentally verified, demonstrating the reliability of the MD method [24,25,26]. In recent years, some attempts have been made to perform MD simulations of silicon-containing arylacetylene resins. The pyrolysis and antioxidant mechanisms of PSA-type resins were systematically investigated by the reactive force field (ReaxFF) MD method [27,28]. Li et al. [29] conducted a study on PSA resins with various chain backbone structures by MD simulations. The interactions between the PSA resins were typical face-to-face π–π stacking interactions, and the viscosity and heat resistance of the resins could be improved by increasing the proportion of aromatic rings. Zhu et al. [30] and Zhang et al. [31] developed a material genetics method that allowed for screening novel silicone-containing arylacetylene resins by defining the “resin genes” and identifying key features of the desired properties. A comprehensive approach combining MD and experimental studies further validated the excellent processability and heat resistance of the screened PSNP and PSNP-MV resins. Moreover, PSNP-MV resin has lower viscosity and higher thermal stability at low temperatures than PSNP resin. Moreover, their molecular structures can be distinguished by the silicon atom linked to the vinyl group in the PSNP-MV structure, suggesting that the vinyl group plays an important role in improving its properties.

**Figure 1:**
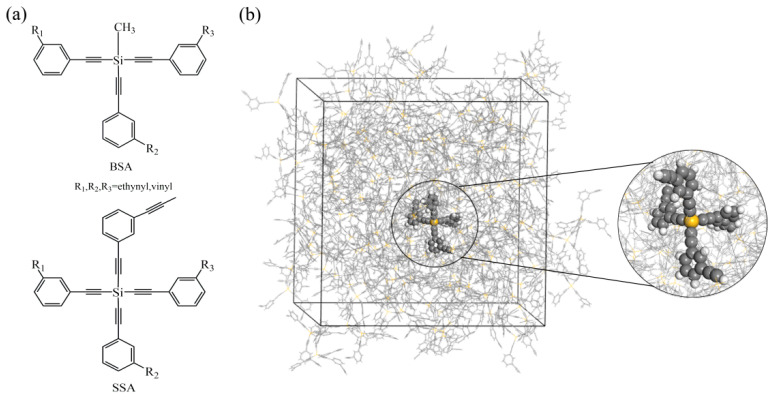
(**a**) Molecular structures of BSA and SSA resins, (**b**) equilibrium model of SSA1 resin.

In order to understand the excellent performance of the vinyl group in high-temperature-resistant resins, it is necessary to explore the relationship between resin properties and vinyl content. This study focuses on two types of branched resins: star-shaped silicon-containing arylacetylene (SSA) resin and branched silicon-containing arylacetylene (BSA) resin. A series of branched resins were obtained by varying the ratio of viny/ethynyl on the benzene ring, as illustrated in Figure 1. The diffusion and thermal properties of the resins were investigated by MD simulations, such as the mean square displacements (MSD), diffusion coefficient (*D*), glass transition temperature (*T*_g_), and the coefficient of linear thermal expansion (CLTE). The effects of temperature on the molecular structure and kinetic properties were elucidated. Finally, the intrinsic relationship between the structure and properties of the resins was thoroughly evaluated.

## 2. Simulation Details

All simulations were performed using Materials Studio 7.0 software [32]. COMPASS (Condensed-phase Optimized Molecular Potentials for Atomistic Simulation Studies) force field was chosen to describe the total potential, including both bonded interaction and non-bonded interaction forms [33]. The COMPASS force field has been successfully validated and predicted for a variety of molecular and structural properties of polymers, and has also been applied to interactions in high-performance thermosetting resins [6,34].

Two-hundred BSA or SSA resin molecules were randomly distributed in a cubic simulation box by using the Amorphous Cell module. Table 1 lists the specifics of all systems, including the ratios of vinyl/ethynyl groups in the substituent of the resin system. The initial density was set to 0.5 g/cm^3^ to eliminate ring spearing and close contacts between resin monomers during the simulation. The van der Waals force was determined by the atom-based summation method with a cutoff distance of 12.5 Å. The Forcite module was used for geometry optimization and MD simulations of the BSA and SSA resins. The Andersen thermostat and Berendsen barostat were used to control the temperature and pressure, respectively. The time step was set to 1.0 fs.

**Figure 2:**
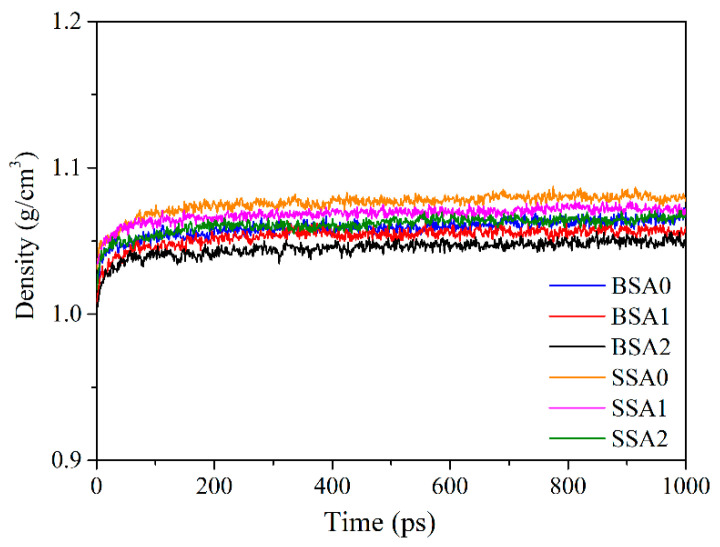
Time evolution of the densities of the BSA and SSA resins.

The initial model was simulated using an isothermal–isobaric (NPT) ensemble through relaxation, annealing, and equilibrium processes. Specifically, the initial model was relaxed at 800 K and 1 atm for 500 ps and then annealed to 300 K. This stage was repeated five times to reduce high internal stresses in the system. Subsequently, the duration of the equilibrium process at 300 K was set to 1 ns to achieve a constant density. Figure 2 shows the density variation of the BSA and SSA resins during the equilibrium process. The densities of the BSA and SSA resins obtained here ranged from 1.053 to 1.079 g/cm^3^, which is similar to those of the dendritic poly (methylsilane arylacetylene) (DPSA-H) and poly (methylsilane arylacetylene) (PSA-H) resins reported by Lv et al. [16]. The results indicate that the final density decreased with increasing vinyl content in the resin, which demonstrates that an increase in the vinyl group can create larger voids between branched resins. Finally, the model was used to run MD simulations for 500 ps under canonical (NVT) ensemble at 300 K to obtain a low-energy configuration. The final configuration was used to calculate free volume, diffusion, and thermal behaviors.

To calculate the properties of the resins, MD simulations were run three times independently for each system. The free volume was simulated under NVT ensemble for 2000 ps, and the last 10 ps was used to calculate a fractional free volume. Diffusion simulations were carried out with NVT ensemble at 400, 500, 600, 700, and 800 K for 2000 ps. The 10 ps time interval was used for sampling and analyzing MSD. The diffusion characteristics of the resins can be evaluated by means of MSD and D. The MSD Equation expresses the offset of the mean position of the model particle relative to the initial position during the simulation time, which, by default, uses the center of mass of the group of atoms in the structure. According to the Einstein equation, *D*(*t*) is proportional to the slope of the MSD-*t* curve at a certain moment and is defined as follows:

(1) $$D(t)=\frac{MSD(t)}{6t}=\frac{\langle {\left|r_{i}(t)-r_{i}(0)\right|}^{2}\rangle }{6t}$$

where *r_i_*(*t*) represents the position of the *i*th molecule at the diffusion time (*t*), and *r_i_*(0) is the initial position of the *i*th molecule. Then, the temperature dependence of the diffusivity can be derived by Arrhenius’s relation shown as:

(2) $$ln(D)=ln(D_{0})-E_{a}/RT$$

where *D*_0_ is the pre-exponential factor of diffusion, *E*_a_ is the diffusion activation energy, *R* is the gas constant, and *T* is the temperature.

For the analysis of thermal properties, MD simulations were carried out using the NPT ensemble in a temperature range of 800–400 K, with a cooling rate of 20 K/ns, and a pressure maintained at 1 atmosphere. To determine the *T*_g_ and CLTE values, the average density (*ρ*) and volume (*V*) were obtained from the last 100 ps run at each temperature. Then, *T*_g_ was located by the inflection point of the volume–temperature (*V*-*T*) fitted straight line, while CLTE (*α*) was calculated by linearly fitting the volume–temperature (*V*-*T*) curve.

## 3. Result and Discussion

### 3.1. Free Volume

According to the free volume theory [35], the presence of free volume ensures space for molecular diffusion and facilitates the fluidity of the resin. The fractional free volume (FFV) is the free volume between resin molecules as a proportion of a specific volume, and can be calculated from the following equation:

(3) $$FFV=\frac{V_{f}}{V_{f}+V_{0}}\times 100\%$$

where *V*_f_ is the free volume and *V*_0_ is the occupied volume.

The FFVs of the BSA and SSA resins were evaluated to understand the effects of free volume on diffusivity. The volume information (*V*_0_, *V*_f_, and FFV) at 400 K and FFV growth rates at 400–800 K for the BSA and SSA resins are presented in Table 2. The main difference between the two types of resins is the molecular spatial configuration. This suggests that a larger branched configuration contributes to an increase in the free volume of the resin [35]. Furthermore, the FFV of the branched resin decreased slightly with the introduction of the substituent vinyl group, resulting in a smaller free space of the resin. For example, the FFVs of BSA0, BSA1, and BSA2 were 17.2%, 16.9%, and 16.7% respectively. This may be attributed to the change in the proportion of vinyl/ethynyl groups, which, on the one hand, increased the molecular weight and decreased the density of the BSA and SSA resins (as shown in Table 1). On the other hand, it also enhanced the intermolecular interactions, which affected the stacking mode of the chain segments and further shrank the BSA and SSA resins.

**Figure 3:**
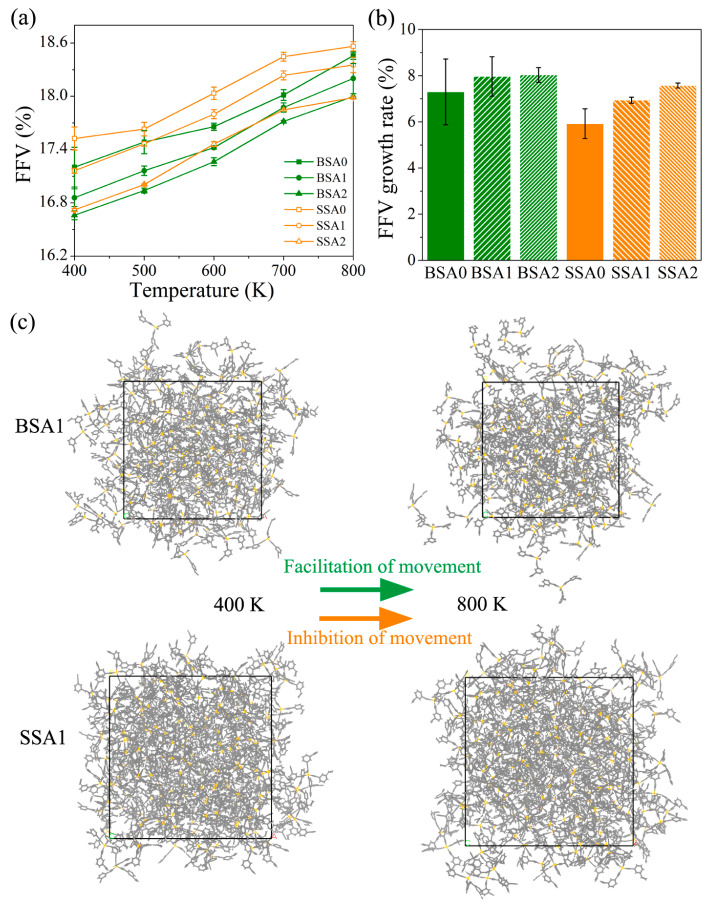
(**a**) FFV versus temperature curves, (**b**) FFV growth rates in a temperature range of 400–800 K for the BSA and SSA resins, and (**c**) snapshots of the BSA1 and SSA1 resins at 400 K and 800 K.

In general, the free volume is greatly dependent on the temperature. As shown in Figure 3a, the FFV of the resin increased with increasing temperature. As the temperature rose, the fluidity of the resin molecules improved and the intermolecular space became larger; thus, FFV increased. More importantly, the FFV growth rates of the SSA resins over a temperature range of 400–800 K were smaller than those of BSA resins (shown in Figure 3b). As seen from the snapshots of the BSA1 and SSA1 resins at 400 K and 800 K, the SSA1 resin exhibited a more compact aggregated chain structure under high-temperature stimuli, relative to the loose structure of the asymmetric BSA1 resin. These findings reflect that the symmetric star-shaped SSA resins are more resistant to deformation at high temperatures, which inhibits the movement of SSA molecules in the voids. As a result, the SSA resins have smaller moving voids, and the addition of the vinyl effectively reduces the free volumes of the resins.

### 3.2. Diffusion Ability

**Figure 4:**
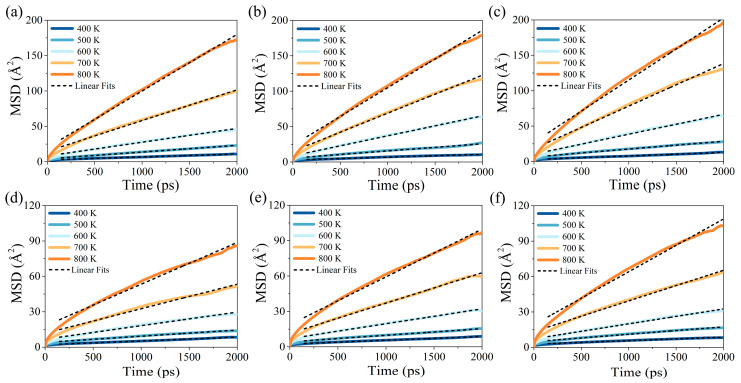
MSD curves for the (**a**) BSA0, (**b**) BSA1, (**c**) BSA2, (**d**) SSA0, (**e**) SSA1, and (**f**) SSA2 resins at different temperatures.

In order to study the resin flow, the diffusion behaviors of the branched resins under different temperatures were calculated. Analyses of mean square displacements and the diffusion coefficient were used to explore the diffusion ability of BSA and SSA resins, which is related to the rheological properties of the branched resins. Figure 4 plots the MSD versus time at 400–800 K for BSA0, BSA1, BSA2, SSA0, SSA1, and SSA2. It can be found that the MSD values of the branched resins exhibited small changes at 400–500 K. The MSD curves showed an evident linear ramp up above 600 K. This may have been due to the fact that higher temperatures increased the fractional free volume, which made the resin molecules diffuse more easily, as verified by the above free volume results. From the MSD curves in Figure 4a,d, it can be observed that the two branched resins (BSA0 and SSA0 resins) showed different mobilities. The slower diffusion rate of the SSA0 resin was attributed to the higher molecular weight of the system and the presence of a symmetrical reactive group, the phenylethynyl group, which was significantly larger than the methyl group of the BSA0 resin, thus hindering the movement of the SSA0 resin molecules and reducing their mobility. Therefore, the smaller the molecular geometry and molecular weight, the faster the diffusion. In addition, the MSD values of the BSA and SSA resins at different vinyl contents were compared. The ranking of MSD values at 800 K was BSA2 > BSA1 > BSA0. It is interesting to note that there was a significant positive effect on the MSD values with the increase in the vinyl content.

The diffusion simulations lasted for 2000 ps: the initial 150 ps was for system equilibration, and 150–2000 ps was for the calculation of diffusion coefficients. It could be found that the time range of 150–2000 ps for the resin was long enough to apply the Einstein relation to determine the diffusion coefficients by the linear fit of the MSD(t) curve. As shown in Figure 4, the R^2^ values of the fitted MSD curves for BSA and SSA resins were greater than 0.98, indicating that MSD(t) is proportional to t, consistent with linear behavior. In order to specifically understand the diffusion behaviors of the six branched resins, the diffusion coefficients of the branched resins at different temperatures are summarized in Table 3. At the same temperature, the SSA resins had lower diffusion coefficients compared with those of the BSA resins. For example, at a temperature of 400 K, the *D* value of the BSA0 resin (6.90 × 10^−8^ cm/s^2^) was slightly higher than that of the SSA0 resin (5.10 × 10^−8^ cm/s^2^). When the temperature was raised to 800 K, the *D* values of BSA0 (1.40 × 10^−6^ cm/s^2^) and SSA0 (6.85 × 10^−7^ cm/s^2^) differed by an order of magnitude. This demonstrates that the branched resins with well-defined symmetry affected the molecular motion and reduced the diffusion coefficient. As reported by Jagarlapudi et al. [36], the higher the molecular weight, the more branched the isomeric structure and the lower the diffusion rate, so the diffusion coefficient decreases.

**Figure 5:**
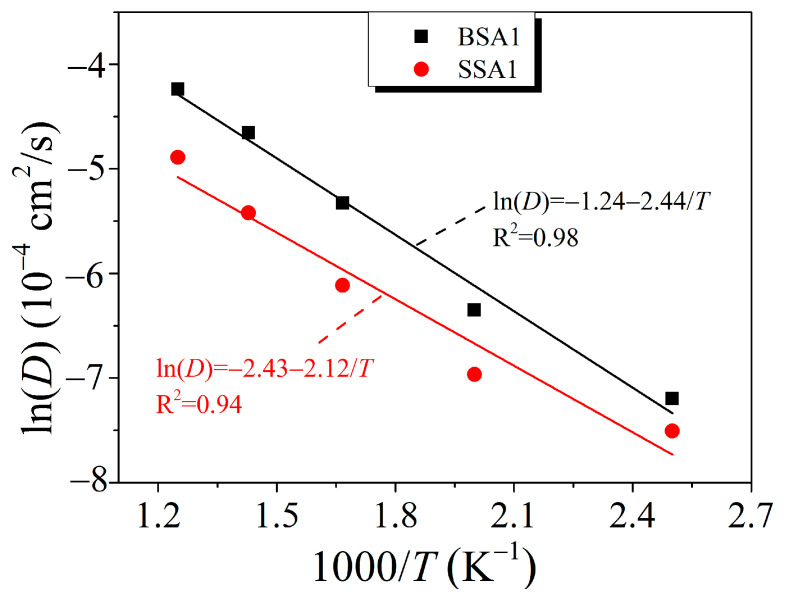
Arrhenius plot of the diffusion coefficients for the BSA1 and SSA1 resins at different temperatures.

Moreover, the ordering of *D*_0_ and *E_a_* is essential to understand the rheological behavior of branched resins. The Arrhenius plot of the diffusion coefficients for the BSA1 and SSA1 resins is shown in Figure 5. The parameters *D*_0_ and *E_a_* of the six branched resins, obtained by fitting Equation (2), are shown in Table 3. The instantaneous diffusion coefficient (*D*_0_) [37] ranked as SSA0 < SSA1 < SSA2 < BSA0 < BSA1 < BSA2. It can be seen that increasing the proportion of the vinyl/ethynyl groups effectively promoted the physical diffusivity of the resin molecules. The variation trend of *E*_a_ was consistent with that of *D*_0_, and SSA resins had s lower diffusion activation energy. Therefore, the branched resins with a symmetric star-shaped structure are insensitive to temperature during diffusion, as evidenced by the smaller FFV growth rate of the SSA0 resin mentioned above. Moreover, Li et al. [29] investigated the rheological and thermal behaviors of linear PSAs, including PSA-V, PSVP-V, and PSA-VF, based on molecular dynamics simulations. The results showed that the diffusion activation energies of these three resins were in the range of 13.30–15.88 kJ/mol, whereas those of the branched BSA and SSA resins were in the range of 17.35–20.52 kJ/mol. The reason for these differences is that the branched resins had higher molecular weights and interaction forces, which needed to cross a larger energy barrier for diffusion. On the other hand, this result also demonstrates that the incorporation of the vinyl group into the branched structure is very sensitive to the temperature and needs to overcome a higher energy barrier, as shown for BSA0, BSA1, and BSA2. Through comprehensive analysis, the larger the molecular weight and spatial configuration of SSA resin, the smaller the diffusion rate. In addition, the addition of the vinyl can accelerate the movement and diffusion of resin molecules and reduce the viscosity of resin, thus improving the rheological properties of resin.

### 3.3. Thermal Performance

It is widely accepted that an increase in crosslink density can reduce the mobility of molecules, thereby increasing the thermal resistance and modulus of resin. In addition, intermolecular forces and star-like topology contribute to the thermal resistance and modulus of the resin [15,16]. The glass transition temperature is an essential indicator to measure the thermal properties of high-performance resin materials, which determines their working temperature and application areas. Interestingly, *T*_g_ is also related to the processing window [38,39]. Chen et al. [40] suggested that uncured resins with lower *T*_g_ and higher cure temperatures could be processed over a wider processing window. Therefore, in this work, the *T*_g_ values of the uncured BSA and SSA resins were calculated and the effects of the vinyl/acetylene-based ratio on the processing window and thermal resistance of the resins were discussed.

**Figure 6:**
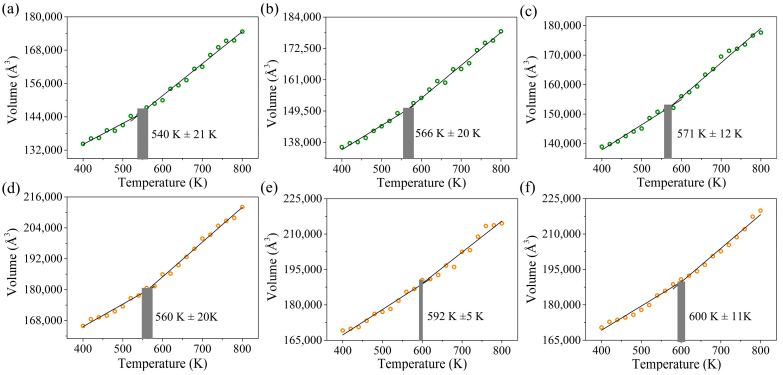
Volume as a function of temperature for (**a**) BSA0, (**b**) BSA1, (**c**) BSA2, (**d**) SSA0, (**e**) SSA1, and (**f**) SSA2.

Figure 6 shows the volume distribution for BSA0, BSA1, BSA2, SSA0, SSA1, and SSA2 at different temperatures. The volume of the six resins increases with the temperature, where the temperature at the intersection point of the two curves is regarded as the *T*_g_ value. As expected, the *T*_g_ values of the SSA resins were higher compared with those of the BSA resins. For example, the *T*_g_ values of BSA1 and SSA1 were 566 ± 20 K and 592 ± 5 K, respectively. In general, the *T*_d5_ and *T*_g_ of heat-resistant resins are positively correlated [30,31]. For example, the *T*_d5_ and *T*_g_ values of SSA1 resin were higher than those of BSA1, which was in agreement with the trend of the experimental results [15]. Moreover, the experiments demonstrated a larger processing window for the smaller *T*_g_ of BSA1 resin compared with that of SSA1 resin. This suggests that a larger symmetrical branched structure can improve the heat resistance, which is because this structure hinders the movement and diffusion of molecular chain segments, increasing the rigidity of the system and making the structure more aggregated. Excitingly, the change in the vinyl/acetylene group ratio did not result in a decrease in *T*_g_, with values of 560 ± 20 K, 592 ± 5 K, and 600 ± 11 K for SSA0, SSA1, and SSA2, respectively. The improvement in the *T*_g_ value was due to the possibility of π–π stacking, in which the introduction of vinyl groups into the resin molecules can enhance the intermolecular interactions and reduce chain spacing.

**Figure 7:**
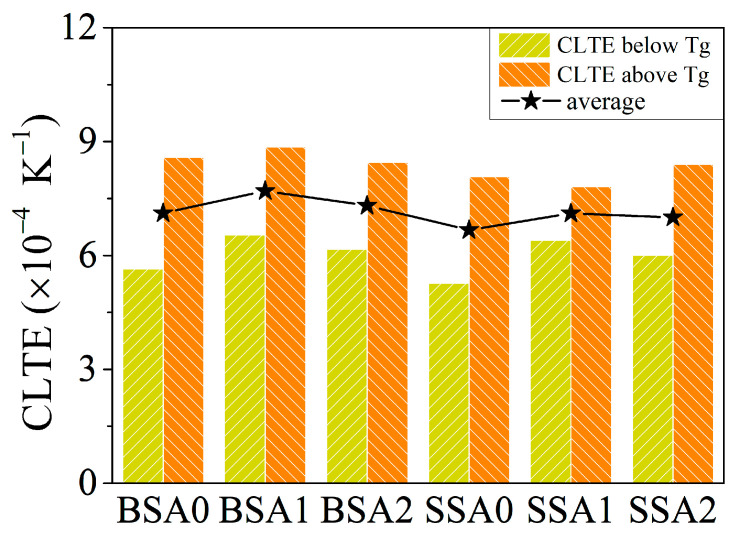
Linear thermal expansion coefficients values of the studied resins.

The coefficient of thermal expansion (CTE) reflects the dimensional stability of amorphous materials during heating/cooling. High-performance resin with a small CTE can effectively prevent large deformation and failure during excessive temperature changes. Researchers have usually changed the resin structure or blend modification to reduce the CTE value [41,42]. Figure 7 displays the CLTE values of the studied resins. Among them, the CLTE values below *T*_g_ were significantly lower than those above *T*_g_. Based on the average CLTE values of various branched resins, it was found that the CLTE value of the SSA0 resin was slightly lower than that of the BSA0 resin. This may also be attributed to the stiffness of the SSA resins and the enhanced intermolecular interactions that hindered the expansion of the molecular chains. Therefore, it may be inferred that the introduction of the vinyl group can enhance the intermolecular interactions, resulting in a tighter molecular stacking of the branched resin and better high-temperature resistance.

### 3.4. Molecular Properties and Interactions

**Figure 8:**
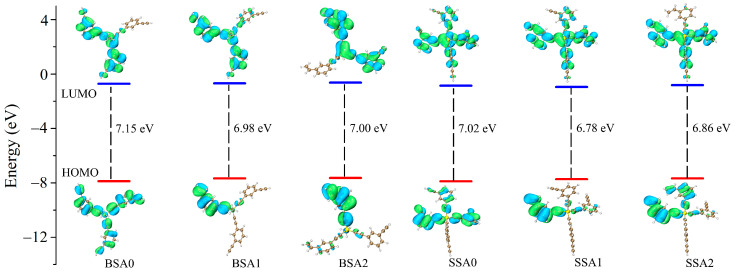
HOMO and LUMO energy levels of the BSA0, BSA1, BSA2, SSA0, SSA1, and SSA2 resins.

The curing temperature and reactivity of resin can be assessed by key electronic features (highest occupied molecular orbital, HOMO, and lowest unoccupied molecular orbital, LUMO) [30]. Previous studies have shown that the lower the |HOMO-LUMO| value, the lower the cure temperature, and the higher the reactivity [6]. The geometries of the resin molecules and the frontier molecular orbitals have been calculated at the DFT-M06-2X/def2-SVP level [43]. The |HOMO-LUMO| values of the BSA0, BSA1, BSA2, SSA0, SSA1, and SSA2 resins were 7.15, 6.98, 7.00, 7.02, 6.78, and 6.86 eV, respectively, as shown in Figure 8. The energy gaps of the SSA resins were lower than those of the BSA resins due to the enhancement in the electron cloud density of the phenylacetylene unit. With the addition of the vinyl group, the resins also had relatively lower energy gaps and similar fragment molecular orbital (FMO) surfaces. The HOMO orbitals were predominantly distributed on the vinyl-connected benzene ring, while the electron density of the LUMO orbitals was delocalized throughout the conjugated unit. This suggests that the SSA1 and SSA2 resins have higher curing reactivity and can only cure at lower temperatures.

**Figure 9:**
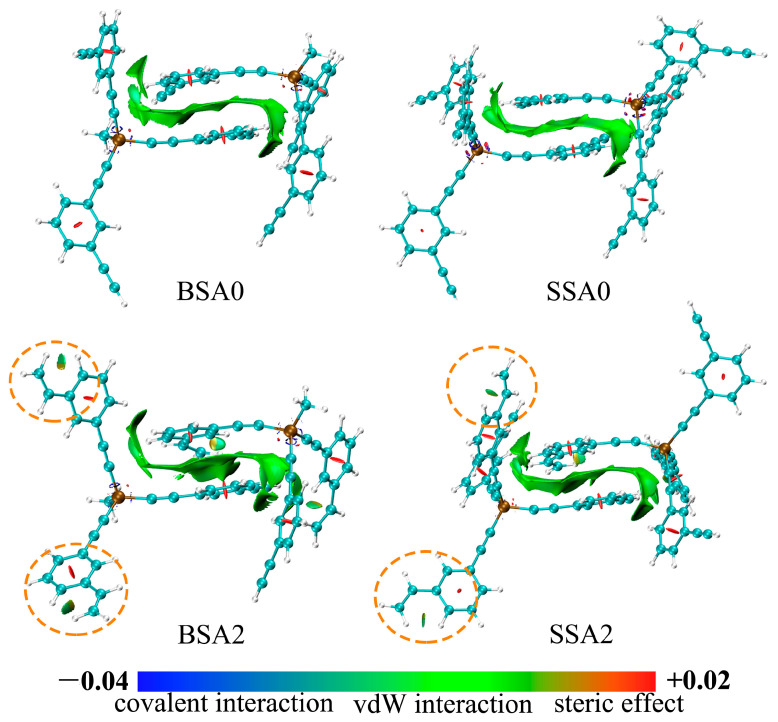
IRI isosurface maps for the BSA0, SSA0, BSA2, and SSA2 resins.

In addition, the interaction region indicator (IRI) can be used to study the covalent and non-covalent interactions of branched resins based on the stacking conformation of equilibrium simulations [44]. According to the coloring criteria of IRI, interactions can be classified into chemical bonds (blue), van der Waals interactions (green), and steric hindrance (red). Figure 9 illustrates the IRI isosurface maps of resin–resin structures obtained using Multiwfn 3.8 and VMD software 1.9.3 [45,46]. Evidently, the IRI isosurface maps of the respective stacking conformations of the BSA and SSA resins were similar in shape, with flat van der Waals interaction regions, which are typical of π–π stacking interactions [29]. The IRI isosurfaces in the center of the benzene ring region had a distinct red state, which indicated the presence of steric hindrance. It is noteworthy that the IRI isosurfaces of BSA2 and SSA2 showed corresponding green flaky isosurfaces around the vicinity of the vinyl groups, marked by yellow circles, reflecting the formation of van der Waals interactions in these regions. This more intuitively explains the increase in the stacking interactions of the BSA and SSA resins after the introduction of the vinyl groups.

In order to quantify the stacking interactions, the stacking interaction energies of the branched resins were calculated by the MD and DFT methods, as shown in Table 4. It is evident that the interaction energies obtained by DFT had the same trend as those calculated by the MD method. The molecular configuration and molecular weight of the symmetric star-shaped SSA resin were larger than those of the BSA resin, resulting in an overall increase in FFV and density along with increased inter-molecular interaction energies for SSA resins compared with BSA resins. The smaller increase in the FFV growth rate for SSA resins also stemmed from their stronger inter-molecular interactions and the formation of a compact chain structure. In addition, BSA0 and SSA0 had the largest free volume fractions and densities with the corresponding smallest interaction energies. Upon incorporation of vinyl groups, the density and fractional free volume decreased and the favorable intermolecular energies increased.

**Figure 10:**
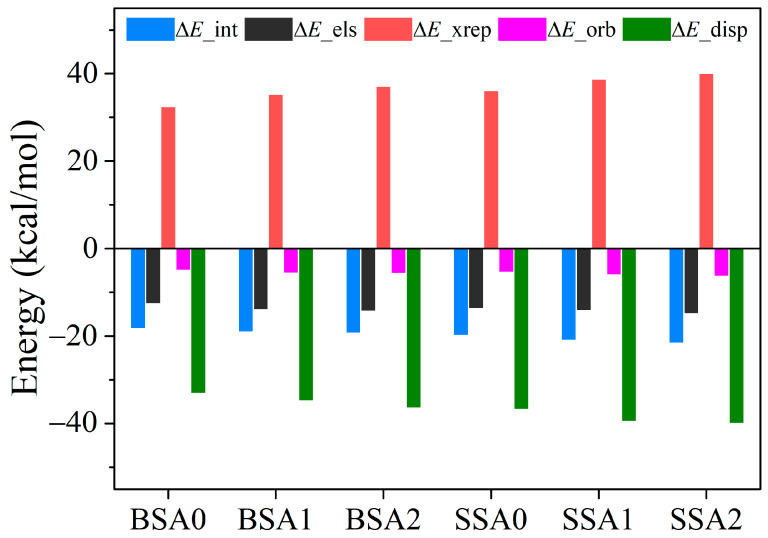
Intermolecular interaction energies (in kcal/mol) for the BSA0, BSA1, BSA2, SSA0, SSA1, and SSA2 resins.

Based on the DFT method, we calculated the energy composition of the molecular stacking interactions of the resins using the energy decomposition method developed by Lu et al. [47]. The interaction energy (Δ*E*_int) consists of electrostatic (Δ*E*_els), exchange repulsion (Δ*E*_xrep), orbital interaction (Δ*E*_orb), and dispersion correction (Δ*E*_disp). The calculated energies are shown in Figure 10 and the specific values are listed in Table 5. In terms of the decomposition energy components, the intermolecular stacking mainly relies on the dispersion effect, followed by electrostatic interactions. With the addition of vinyl, the repulsion and dispersion effects increased significantly. Therefore, the high-temperature resistance of the resin can be improved by modulating the π–π stacking interactions through targeted changes in the resin structure.

### 3.5. Comprehensive Comparison of Performance

**Figure 11:**
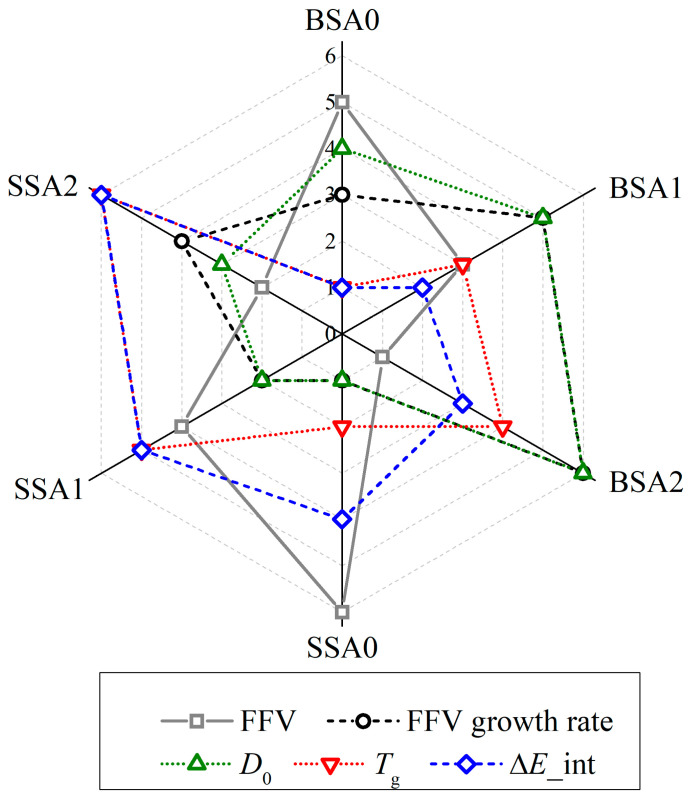
Performance radar chart and scores of the BSA0, BSA1, BSA2, SSA0, SSA1, and SSA2 resins.

After the above analysis, the effects of different vinyl contents on the diffusion and thermal properties of the branched resins were disclosed. In order to comprehensively evaluate and rank the properties of the six branched resins, FFV, FFV growth rate, *D*_0_, *T*_g_, and Δ*E*_int were selected as the key indexes and a radar chart was plotted to improve the visualization of the results. A larger diffusion coefficient indicates a better rheological performance of the PSA resin, while a higher glass transition temperature indicates better heat resistance. A radar chart allows for the calculated property values or absolute values of the property values to be ranked in increasing order, and each best property is assigned to the system by indicating the corresponding maximum value. That is, the best performance is given as 6 scores, followed by 5, 4, 3, and 2, and the lowest performance is assigned to a score of 1. Figure 11 shows the radar chart and scores for the properties of the six branched resins. The positive correlation between interaction energy and thermal properties was also verified, indicating that an increase in the weak intermolecular interactions of the resins can improve high temperature resistance. And the rheological properties were more correlated with the FFV growth, rate rather than the FFV value. The thermal property scores of the SSA resins were significantly higher than those of the BSA resins, while the diffusion property scores of the BSA resins were relatively high. In addition, the advantages of diffusion and thermal properties were more pronounced as the number of the vinyl group increased.

## 4. Conclusions

In this study, two types of branched silicon-containing arylacetylene resins, BSA and SSA, were investigated using MD simulations, and the effects of the vinyl/ethynyl group ratio on their diffusion and thermal properties were analyzed. The simulation results show that minor differences in the molecular structure of the branched resins can significantly affect their properties. First, the unique branched structure of SSA0 resin exhibited a larger free volume fraction compared with the BSA0 resin. But the incorporation of the symmetrical phenylethynyl group increased the rigidity of the chain segments and inhibited the diffusion ability of the resin, which improved the heat resistance. Secondly, the increase in the vinyl/ethynyl group ratio positively affected both the rheological properties and heat resistance of the resin. This may be due to the fact that branched resins exhibit stronger intermolecular interactions, which are dominated by dispersion effects, and the addition of the vinyl groups correspondingly increases intermolecular repulsions. In conclusion, the combination of a well-defined symmetric branched structure with vinyl groups can effectively improve the high-temperature resistance of branched resins. This insight is valuable for controlling the thermal properties of PSA resins through strategic structural design.

## Figures and Tables

**Table 1:** Substituent, number of atoms, and *ρ* of the systems.

Systems	Substituent			Number of Atoms	*ρ* (g/cm^3^)
	R_1_	R_2_	R_3_		
BSA0	+	+	+	10,000	1.067
BSA1	+	+	−	10,400	1.059
BSA2	+	−	−	10,800	1.053
SSA0	+	+	+	12,200	1.079
SSA1	+	+	−	12,600	1.071
SSA2	+	−	−	13,000	1.070

Note: “+” represents ethynyl group, “−” represents vinyl group.

**Table 2:** Volume information (*V*_0_, *V*_f_, and FFV) at 400 K and FFV growth rates at 400–800 K for the BSA and SSA resins.

Models	*V*_0_ (Å^3^)	*V*_f_ (Å^3^)	FFV (%)	FFV Growth Rate (%)
BSA0	107,889.00 ± 92.12	22,415.87 ± 92.11	17.20 ± 0.22	7.30 ± 1.42
BSA1	109,649.15 ± 77.89	22,223.40 ± 77.09	16.86 ± 0.10	7.97 ± 0.85
BSA2	111,089.49 ± 65.75	22,207.40 ± 65.34	16.66 ± 0.05	8.03 ± 0.32
SSA0	134,187.28 ± 72.15	28,513.16 ± 72.27	17.52 ± 0.13	5.92 ± 0.65
SSA1	136,285.50 ± 34.01	28,235.89 ± 34.01	17.16 ± 0.03	6.94 ± 0.13
SSA2	137,061.64 ± 27.34	27,991.85 ± 27.35	16.72 ± 0.02	7.57 ± 0.11

**Table 3:** Diffusion coefficients (*D*), pre-exponential factors (*D*_0_), and diffusion activation energies (*E*_a_) for the BSA and SSA resins at different temperatures.

Resins	*D* (×10^−8^ cm/s^2^)					*D*_0_ (×10^−4^ cm/s^2^)	*E_a_* (kJ/mol)
	400 K	500 K	600 K	700 K	800 K		
BSA0	6.90 ± 0.39	15.78 ± 0.11	34.50 ± 0.41	75.80 ± 0.08	140.1 ± 1.95	0.23 ± 0.14	19.89 ± 2.17
BSA1	7.48 ± 0.04	17.47 ± 0.05	48.47 ± 0.20	94.90 ± 1.77	144.4 ± 1.75	0.29 ± 0.05	20.26 ± 0.38
BSA2	7.97 ± 0.63	19.62 ± 0.16	49.85 ± 0.09	109.4 ± 1.02	159.7 ± 1.23	0.33 ± 0.12	20.52 ± 2.48
SSA0	5.10 ± 0.68	9.92 ± 0.91	21.87 ± 0.03	40.90 ± 0.10	68.50 ± 0.58	0.08 ± 0.01	17.35 ± 0.93
SSA1	5.48 ± 0.04	9.72 ± 0.20	22.13 ± 0.15	44.18 ± 0.56	75.13 ± 1.99	0.09 ± 0.002	17.58 ± 0.08
SSA2	5.62 ± 0.62	11.75 ± 0.3	23.15 ± 0.07	48.42 ± 3.26	85.70 ± 0.05	0.10 ± 0.01	17.94 ± 0.66

**Table 4:** Stacking interaction energies (in kcal/mol) for the BSA0, BSA1, BSA2, SSA0, SSA1, and SSA2 resins obtained by MD and DFT method.

	Δ*E*_int _(MD)_	Δ*E*_int _(DFT)_	Average Difference (%)
BSA0	−20.29	−18.34	5.05
BSA1	−21.35	−19.10	5.56
BSA2	−21.78	−19.36	5.88
SSA0	−23.04	−19.88	7.36
SSA1	−23.16	−21.02	4.84
SSA2	−25.37	−21.67	7.87

Note: $Average\;difference=\frac{\Delta E\_int(MD)-\left[\Delta E\_int(MD)+\Delta E\_int(DFT)\right]/2}{\left[\Delta E\_int(MD)+\Delta E\_int(DFT)\right]/2}\times 100\%$.

**Table 5:** Energy composition of stacking interaction energies (in kcal/mol) for the BSA0, BSA1, BSA2, SSA0, SSA1, and SSA2 resins.

	Δ*E*_els	Δ*E*_xrep	Δ*E*_orb	Δ*E*_disp
BSA0	−12.64	32.46	−5.03	−33.13
BSA1	−14.01	35.34	−5.63	−34.80
BSA2	−14.32	37.1	−5.68	−36.47
SSA0	−13.79	36.18	−5.46	−36.82
SSA1	−14.19	38.77	−6.07	−39.54
SSA2	−14.93	40.06	−6.37	−40.03

## Data Availability

Data are contained within the article.
